# Validating image-derived input functions of dynamic ^18^F-FDG long axial field-of-view PET/CT studies

**DOI:** 10.3389/fnume.2025.1556848

**Published:** 2025-02-28

**Authors:** Charlotte L. C. Smith, Gerben J. C. Zwezerijnen, Marijke E. den Hollander, Henricus N. J. M. Greuter, Nienke R. Gerards, Josée Zijlstra, C. Willemien Menke-van der Houven van Oordt, Idris Bahce, Maqsood Yaqub, Ronald Boellaard

**Affiliations:** ^1^ Department of Radiology and Nuclear Medicine, Amsterdam UMC Location Vrije Universiteit Amsterdam, Amsterdam, Netherlands; ^2^ Imaging and Biomarkers, Cancer Center Amsterdam, Amsterdam, Netherlands; ^3^ Department of Hematology, Amsterdam UMC Location Vrije Universiteit Amsterdam, Amsterdam, Netherlands; ^4^ Department of Medical Oncology, Amsterdam UMC Location Vrije Universiteit Amsterdam, Amsterdam, Netherlands; ^5^ Department of Pulmonary Medicine, Amsterdam UMC Location Vrije Universiteit Amsterdam, Amsterdam, Netherlands

**Keywords:** IDIF, LAFOV PET, ^18^F-FDG, dynamic PET imaging, non-invasive quantification

## Abstract

**Aim/background:**

Dynamic PET imaging requires an input function typically obtained through blood sampling. Image-derived input functions (IDIFs) of the ascending aorta (AA), aortic arch, descending aorta (DA), or left ventricle (LV) offer non-invasive alternatives, especially with long-axial field-of-view (LAFOV) PET/CT systems enabling whole-body dynamic ^1^⁸F-FDG imaging. This study aimed to validate uncorrected IDIFs derived from the AA, DA, aortic arch, and LV by comparing them to (late) venous whole-blood in patients undergoing LAFOV PET/CT.

**Methods:**

Eleven oncology patients who underwent 70-min dynamic ^18^F-FDG PET/CT scans on a LAFOV PET/CT system after receiving an intravenous bolus injection of 3.0 MBq/kg were included. Seven venous blood samples were collected manually at approximately 5, 10, 15, 25, 35, 45, and 60 min post-injection (pi) and compared to IDIFs derived from the AA, aortic arch, DA, and LV. Bias between IDIFs and venous blood samples was assessed at each time point.

**Results:**

IDIF accuracy relative to venous blood samples improved over time, with a median percentage bias <10% after 25 min pi. At 60 min pi, the aortic arch showed the smallest bias (median −1.1%, IQR 5.9%), followed by the AA (2.5%, IQR 7.0%), DA (5.1%, IQR 8.6%), and LV (7.4%, IQR 7.6%).

**Conclusion:**

The high precision of aorta-derived IDIFs suggests that IDIFs are a reliable alternative to manual blood sampling for dynamic ^18^F-FDG PET imaging on a LAFOV PET/CT system. Using IDIFs reduces variability, simplifies protocols, minimizes radiation exposure, and enhances patient safety with a non-invasive approach.

## Introduction

^18^F-fluoro-deoxy-glucose (^18^F-FDG) positron emission tomography-computed tomography (PET/CT) imaging is widely used in oncology (1, 2). Clinical ^18^F-FDG PET imaging often relies on static PET scans, where tracer uptake is primarily assessed by semi-quantitative metrics, such as the standardized uptake value (SUV) (3). However, examining ^18^F-FDG uptake through semi-quantitative metrics is debated since it has several limitations, such as not taking the variation of ^18^F-FDG availability in the blood into account (4–7).

Dynamic PET imaging overcomes many limitations by providing spatiotemporal information on tracer kinetics (8–11). This approach requires an accurate input function, typically obtained through arterial blood sampling (12). Yet, arterial blood sampling is invasive, technically challenging, and associated with potential adverse events, making it preferable to avoid if possible (13–15). Arterialized venous blood sampling or venous blood sampling may be a potential alternative for ^18^F-FDG as there is an arteriovenous equilibrium after approximately 15–30 min (16, 17). However, these approaches still complicate scanning protocols and frequent blood sampling increases the radiation burden for technicians (12). Prior studies on short axial field-of-view (SAFOV) PET/CT systems have demonstrated that, for ^18^F-FDG, an image-derived input function (IDIF) from the aorta or left ventricle (LV) can replace manual blood sampling when assessing whole-blood activity (12, 18, 19). Additionally, Palard-Novello et al. (8) validated IDIFs corrected for plasma-to-whole-blood ratios with venous blood samples when performing dynamic ^18^F-FDG PET scans on a long axial field-of-view (LAFOV) PET/CT system. They suggested that manual blood sample calibration might be unnecessary, given the high precision of the IDIF.

Eliminating the need for manual blood sampling in IDIF calibration would simplify scanning protocols, reduce patient invasiveness, and decrease radiation exposure for personnel. However, the accuracy of whole-blood ^1^⁸F-FDG activity derived from manual blood samples compared to time-activity curves (TACs) from uncorrected IDIFs in LAFOV PET/CT systems remains unclear. Therefore, this study aimed to validate uncorrected IDIFs derived from the ascending aorta (AA), descending aorta (DA), aortic arch, and LV in patients undergoing LAFOV PET/CT. The accuracy of the IDIF was assessed by comparing it with (late) venous blood samples.

## Method

### Study population

Eleven consecutive patients were included from March 2023 to December 2023 at Amsterdam UMC, location VUmc. Each patient underwent a 70-min dynamic ^18^F-FDG PET scan on the Siemens Biograph Vision Quadra PET/CT system (Siemens Healthineers, Knoxville, TN, USA) (LAFOV PET/CT system). Before any study procedures written informed consent was obtained from all patients, and the study was approved by the Medical Ethics Review Committee of the Amsterdam UMC (NL80924.029.2).

### ^18^F-FDG PET/CT acquisition

PET/CT scans were performed and reconstructed according to the European Association of Nuclear Medicine Research Ltd (EARL2) guidelines (20). Each patient received 3.0 MBq/kg ^18^F-FDG at the start of the scan through an intravenous bolus injection. Patients fasted for 4-6 h before tracer administration and plasma glucose levels were <7.0 mmol/L (20). Patients were scanned in one bed position (106 cm), covering the skull vertex to mid-thigh. List-mode PET data started immediately after tracer administration and continued for 70 min. The dynamic PET images were binned in 20 data frames (1 × 15 s, 3 × 5 s, 3 × 10 s, 4 × 60 s, 2 × 150 s, 2 × 300 s, 5 × 600 s). Images were reconstructed using the PSF + TOF OSEM algorithm with 5 subsets, 4 iterations, a matrix size of 220 × 220, slice thickness of 3, voxel size of 3.3 × 3.3 × 2 mm^3^ and a Gaussian filter of 4 mm.

Seven venous blood samples were manually collected via the median cephalic vein or port-a-cath at approximately 5, 10, 15, 25, 35, 45, and 60 min post-injection (pi) and compared with the IDIFs. IDIFs were obtained by manually placing multiple circular-shaped regions of interest (ROI) within the AA, DA, aortic arch, and LV. Ten ROIs were sequentially arranged in the center of each structure, creating a total volume of 1.9602 ml. ROI placement was guided using the early PET frames and the low-dose CT scan, and the same ROIs were then copied to all PET frames. ROI positioning was checked and adjusted if needed to ensure accurate placement in every frame. In cases of PET-CT mismatch, ROIs were realigned based on the PET images.

TACs were generated by projecting the ROIs onto the dynamic PET frames and compared with venous blood samples. Bias between whole-blood activity from the venous blood samples compared to the IDIFs was determined for each time point that the venous blood samples were collected by interpolating the IDIF to the corresponding time. Relative bias for each IDIF at each time point was calculated using the following equation:

$$\begin{array}{rl} \text{\%}\;\mathrm{bias}= & \;((\mathrm{interpolated}\;\mathrm{IDIF}-\mathrm{venous}\;\mathrm{blood}\;\mathrm{sample})/\\ & \;\mathrm{venous}\;\mathrm{blood}\;\mathrm{sample})*100 \end{array}$$

A relative bias below 10% was considered an acceptable agreement between IDIF and venous blood samples and an acceptable agreement was expected after approximately 15–30 min when the arteriovenous equilibrium is established (16, 17). The relative bias is summarized as medians and interquartile ranges (IQR) and represented with Tukey's boxplots.

## Results

### Patient demographics

Eleven oncologic patients (36.4% female) were included in the study. The cohort included four patients with multiple myeloma, one with breast cancer, one with melanoma, one with a Kaposi sarcoma, two with large B-cell lymphoma (DLBCL), one with a T-cell lymphoma and one with a salivary gland tumor. Nobody had a history of type I or II diabetes mellitus. Patient demographics are summarized in Table 1.

**Table 1:** Patient demographics.

Characteristics	Value
Gender (F/M), number	4/7
Age (years), mean (range)	61.8 (48–79)
Length (cm), mean (SD)	177.2 (11.8)
Weight (kg), mean (SD)	80.6 (19.7)
Net injected dose (MBq), mean (SD)	248.2 (62.0)

cm, centimeter; F, female; kg, kilogram; M, male; MBq, mega Becquerel; SD, standard deviation.

### IDIF validation

**Figure 1:**
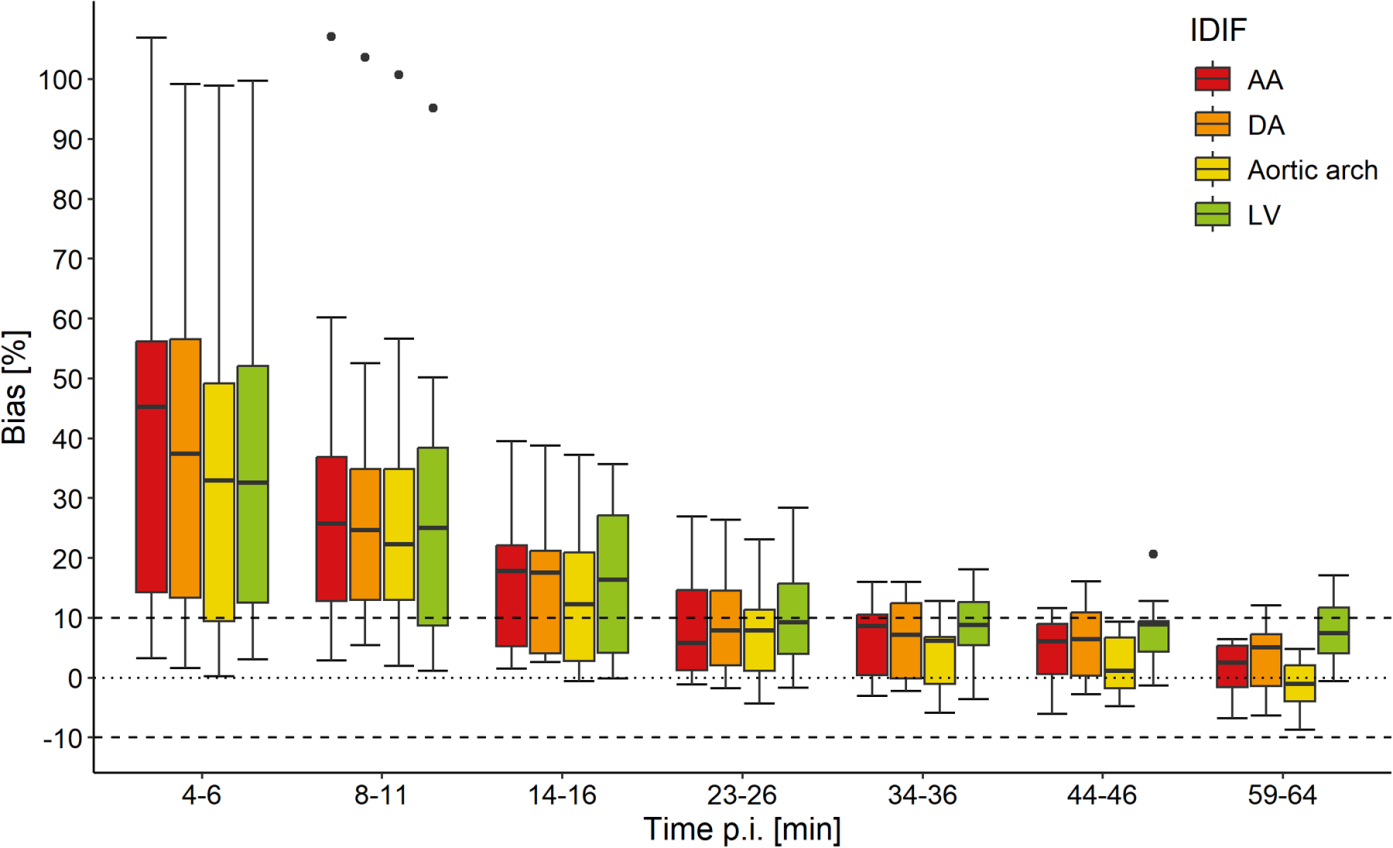
Relative bias in whole-blood ^18^F-FDG activity between venous blood samples and image-derived input functions (IDIFs) of the ascending aorta (AA), descending aorta (DA), aortic arch, and left ventricle (LV) for seven time points post-injection (pi). The horizontal dotted line indicates 0% bias and the two dashed lines indicate −10% and 10% bias. The central line of the box represents the median and the edges are the 25th and 75th percentiles. The extreme data points which are not considered outliers are illustrated with the whiskers.

The accuracy of the IDIFs compared to the venous blood samples improved over uptake time. Substantial differences between the IDIFs and venous blood samples were found in 5, 10, and 15 min pi. The median percentage bias between the venous blood samples and the IDIFs was <10% from 25 min pi and onwards, with the best alignment at 60 min pi. At 25 min pi, the IDIF derived from the AA showed the smallest relative bias compared to the venous blood samples (median 5.8%, IQR 13.3%), followed by the aortic arch (median 7.9%, IQR 10.2%) and DA (median 7.9%, IQR 12.4%). The IDIF derived from the LV showed the largest bias (median 9.2%, IQR 11.7%). At 60 min pi, the IDIF from the aortic arch showed the smallest relative bias compared with venous blood samples (median −1.1%, IQR 5.9%), followed by the AA (median 2.5%, IQR 7.0%), DA (median 5.1%, IQR 8.6%), and LV (median 7.4%, IQR 7.6%). The relative median and IQR for each IDIF compared to the venous blood samples for each time point are summarized in Table 2 and illustrated in Figure 1.

**Table 2:** Relative bias in whole-blood ^18^F-FDG activity between IDIFs and venous blood samples at seven time points post-injection.

Time interval (min pi)	AA	DA	Aortic arch	LV
% Bias (MBq) 4–6 min pi, median (IQR)	45.3 (41.9)	37.4 (43.2)	32.9 (39.8)	32.6 (39.6)
% Bias (MBq) 8–11 min pi, median (IQR)	25.8 (24.1)	24.6 (21.9)	22.3 (21.9)	25.0 (29.7)
% Bias (MBq) 14–16 min pi, median (IQR)	17.8 (16.9)	17.5 (17.2)	12.3 (18.2)	16.4 (22.9)
% Bias (MBq) 23–26 min pi, median (IQR)	5.8 (13.3)	7.9 (12.4)	7.9 (10.2)	9.2 (11.7)
% Bias (MBq) 34–36 min pi, median (IQR)	8.7 (10.2)	7.1 (12.6)	6.2 (7.9)	8.8 (7.2)
% Bias (MBq) 44–46 min pi, median (IQR)	6.1 (8.4)	6.5 (10.6)	1.1 (8.5)	8.9 (5.1)
% Bias (MBq) 59–64 min pi, median (IQR)	2.5 (7.0)	5.1 (8.6)	−1.1 (5.9)	7.4 (7.6)

AA, ascending aorta; DA, descending aorta; IQR, interquartile range; LV, left ventricle; MBq, mega Becquerel; pi, post-injection.

## Discussion

With this study, we aimed to validate the performance of IDIFs obtained from the AA, DA, aortic arch, and LV by comparing them to (late, >30 min p.i.) whole-blood venous blood samples in patients undergoing LAFOV PET/CT imaging.

We observed an acceptable agreement (<10% relative bias) between venous blood and IDIFs from 25 min pi onward, with the best alignment at 60 min pi. Substantial relative bias in whole-blood ^18^F-FDG activity was found between IDIFs and venous blood at 5, 10, and 15 min pi, suggesting that approximately 15 to 25 min were needed to establish an equilibrium in whole-blood ^18^F-FDG activity between venous and arterial blood. The best alignment at 60 min pi is likely due to the distribution of ^18^F-FDG into tissues as a steady-state appears after approximately 60 min (20). Among the evaluated IDIFs, the aortic arch showed the smallest bias relative to venous blood, closely followed by the AA and DA. The superior performance of aorta-derived IDIFs found in this study aligns well with prior research on SAFOV (12, 18, 19) and LAFOV PET/CT systems (8). In contrast, the IDIF derived from the LV exhibited an increased bias, likely due to myocardial spillover, as previously reported by Weerdt et al. (12) and Sari et al. (9) using SAFOV PET/CT systems.

Given the high precision of IDIFs at later time points, our findings suggest that manual blood sampling for IDIF calibration may not be necessary. This would allow IDIFs to serve as the sole input function for dynamic ^18^F-FDG PET imaging. This would make dynamic ^18^F-FDG PET imaging more clinically feasible by simplifying study protocols, reducing invasiveness for patients, and minimizing radiation exposure for personnel (13–15). If IDIF calibration is required, it may be feasible to use venous blood samples collected at later time points instead of arterial blood sampling. While this approach is not entirely non-invasive, it is significantly less invasive and more practical for clinical implementation than arterial blood sampling (13–15). Our results align with the findings of Palard-Novello et al. (8) and support prior SAFOV PET/CT research (12, 18, 19), which indicated that manual blood sampling can be replaced with an aorta-derived IDIF. Additionally, using IDIFs may reduce variability introduced by delays in venous blood sampling, which would otherwise require correction.

A limitation of this study is the substantial relative bias observed during the first 15 min pi. However, this aligns with previous findings (16, 17), which indicate that it takes approximately 15–30 min for arteriovenous equilibrium to be established. Therefore, the early differences observed are likely attributable to physiological disparities between arterial and venous blood rather than inaccuracies in the IDIFs. Had arterial whole-blood samples been available for comparison, these early discrepancies might not have been present. Nevertheless, venous blood sampling was preferred over arterial sampling due to its lower invasiveness and reduced risks such as arterial occlusions and peripheral tissue ischemia (13–15).

In conclusion, venous whole-blood activity showed accurate alignment with IDIFs from 25 min pi onward, with the best alignment at 60 min pi. IDIFs derived from the AA, aortic arch, and DA exhibited the highest precision, whereas the LV showed increased bias, making aorta-derived IDIFs preferable. The high precision of aorta-derived IDIFs suggests that uncorrected IDIFs may serve as a reliable alternative to manual blood sampling in dynamic ^18^F-FDG PET scans conducted on a LAFOV PET/CT system. The use of IDIFs reduces variability, simplifies study protocols, and enhances safety for both patients and personnel, as IDIFs are non-invasive and minimize radiation exposure.

## Data Availability

The datasets generated during and/or analyzed during the current study are available from the corresponding author upon reasonable request.
